# Regulation of Transplanted Cell Homing by FGF1 and PDGFB after Doxorubicin Myocardial Injury

**DOI:** 10.3390/cells10112998

**Published:** 2021-11-03

**Authors:** Mark Baguma-Nibasheka, Tiam Feridooni, Feixiong Zhang, Kishore B.S. Pasumarthi

**Affiliations:** Department of Pharmacology, Dalhousie University, Halifax, NS B3H 4R2, Canada; Mark.baguma-nibasheka@dal.ca (M.B.-N.); Tiam.feridooni@gmail.com (T.F.); Fxzhang@cnu.edu.cn (F.Z.)

**Keywords:** ventricular cell migration, growth factor and chemokine receptors, doxorubicin, cardiomyopathy, cardiac dysfunction

## Abstract

There is no effective treatment for the total recovery of myocardial injury caused by an anticancer drug, doxorubicin (Dox). In this study, using a Dox-induced cardiac injury model, we compared the cardioprotective effects of ventricular cells harvested from 11.5-day old embryonic mice (E11.5) with those from E14.5 embryos. Our results indicate that tail-vein-infused E11.5 ventricular cells are more efficient at homing into the injured adult myocardium, and are more angiogenic, than E14.5 ventricular cells. In addition, E11.5 cells were shown to mitigate the cardiomyopathic effects of Dox. In vitro, E11.5 ventricular cells were more migratory than E14.5 cells, and RT-qPCR analysis revealed that they express significantly higher levels of cytokine receptors *Fgfr1*, *Fgfr2*, *Pdgfra*, *Pdgfrb* and *Kit*. Remarkably, mRNA levels for *Fgf1, Fgf2, Pdgfa* and *Pdgfb* were also found to be elevated in the Dox-injured adult heart, as were the FGF1 and PDGFB protein levels. Addition of exogenous FGF1 or PDGFB was able to enhance E11.5 ventricular cell migration in vitro, and, whereas their neutralizing antibodies decreased cell migration. These results indicate that therapies raising the levels of FGF1 and PDGFB receptors in donor cells and or corresponding ligands in an injured heart could improve the efficacy of cell-based interventions for myocardial repair.

## 1. Introduction

Anticancer drugs such as doxorubicin (Dox), tyrosine kinase inhibitors and various immunotherapies are known to increase disease-free survival rates in the affected patients. However, many of these therapies are associated with cardiotoxicity which makes it challenging for early detection and management of adverse cardiovascular complications in surviving patients [1,2]. In contrast to other cancer therapeutics, Dox is well known to induce apoptosis in the heart and other tissues by inhibiting the actions of Topoisomerase II enzyme and increasing the intracellular levels of reactive oxygen species [3]. Dox-induced cardiotoxicity is frequently associated with several adverse side effects including myocardial ischemia, arrhythmias, impairment of systolic and diastolic functions and heart failure [2,4]. Distinct types of Dox-induced morphological changes were seen during cardiomyocyte loss and vascular degeneration at the ultrastructural level [5]. In addition to these findings, recent animal studies revealed that acute intraperitoneal administration of Dox can cause interstitial and vascular fibrosis in the hearts of recipient mice three days post-injection [3]. The increased fibrosis levels seen in recipient mice could be attributed to increased generation of reactive oxygen and nitrogen species (ROS/RNS), leading to the activation of apoptotic pathways [3].

Myocardial damage caused by Dox cannot be repaired by endogenous mechanisms due to the limited proliferation capacity of cardiomyocytes in the adult heart [6,7]. In addition, recent reports also suggest a minimal role for endogenous stem/progenitor cells in the regeneration of diseased myocardium [6,8,9]. Although conventional heart failure medications and modern cardioprotective treatments such as dexrazoxane and liposome encapsulations are frequently used to prevent Dox induced cardiotoxicity [10], none of these interventions can replace the dead myocardial tissue. To this end, donor cell transplantation is emerging as an exciting option to repair the injured myocardium [6,9,11]. Transplantation of mesenchymal stem cells (MSC) has recently shown a greater promise for the prevention of cardiotoxicity by replacing the injured tissue due to their paracrine effects [12,13,14]. While recent studies confirmed the functional integration and cardioprotective role of transplanted fetal cardiomyocytes (CM; [15,16,17]), the efficacy of such intervention in Dox induced cardiotoxicity has not thoroughly been tested. The discovery of functional coupling between transplanted CMs and host myocardial cells [15,16,17] and the ability of transplanted fetal CMs to confer protection against the induction of arrhythmia [15,17] are significant steps towards the use of CM based therapies for myocardial repair.

Progenitor cells are generally known to migrate to the injured tissues [18,19]. Notably, embryonic ventricles at day 11.5 gestation (E11.5) were shown to harbor a large number of cardiac progenitor cells (CPCs) compared to later stages of development [20]. Moreover, these embryonic CPC exhibit higher cell proliferation capacity when compared to differentiated CM [21]. It was also demonstrated that direct intracardiac injection of E11.5 ventricular cells can form larger grafts when compared to E14.5 ventricular cells due to developmental differences in the relative distribution of CPCs and CMs and their cell cycle activities [21].

The main objective of this study was to compare the cardioprotective effects of ventricular cells obtained from different stages of embryonic development in a mouse model of Dox induced cardiotoxicity. To determine the donor cell ability to mitigate cardiomyopathic effects, we infused E11.5 and E14.5 ventricular cells into the tail veins of Dox treated adult mice and compared the homing efficiencies, ability to induce an angiogenic response and improve myocardial function post cell injection. We recapitulated in vivo donor cell homing data using embryonic ventricular cell cultures in vitro and identified potential candidates and signaling pathways responsible for cell migration.

## 2. Materials and Methods

### 2.1. Chemicals and Reagents

Culture medium, fetal bovine serum (FBS) and all other culture reagents were from Gibco BRL (Burlington, ON, Canada). Recombinant growth factors (rMuFGF1^TM^ Cat#1340-01-10 and rHuPDGFBB^TM^ Cat#1150-10-2) were obtained from Gold Biotechnology (St. Louis, MO, USA), and their antibodies from Santa Cruz Biotechnology (Santa Cruz, CA, USA). All other chemicals were from Sigma-Aldrich Chemical Company (St. Louis, MO, USA) unless otherwise specified.

### 2.2. Experimental Animals

C57BL/6 (BL6) and CD1 mice were obtained from Charles River Laboratories, Montreal, Canada. Generation of mice with Cre recombinase inserted into the Nkx2.5 allele was previously described [22], and the R26R-lacZ reporter strain was obtained from The Jackson Laboratory, Bar Harbor, ME. In the Cre/LoxP system, mice from the Nkx2.5-Cre line (designated NC), are crossed with a Cre-dependent β-galactosidase (lacZ) reporter mouse strain (R26R-lacZ; designated RL). In the resulting double knock-in offspring (designated NCRL), expression of Nkx2.5 results in Cre mediated excision of the floxed stop cassette located in the 5′ region of lacZ sequence and leads to reporter gene expression in cells of the Nkx2.5^+^ lineage. The NCRL embryos were obtained by timed-mating NC and RL parents and noontime on the day the copulation plug was designated embryonic day (E) 0.5 as reported earlier [23,24]. For genotyping, ear punch biopsy genomic DNA was amplified using the Sigma-Aldrich REDExtract-N-Amp^TM^ Tissue PCR Kit and appropriate primer sets for each knock-in line [23,24]. All animal procedures followed the Canadian Council on Animal Care guidelines approved by the Dalhousie University Committee on Laboratory Animal Care (protocols #16-048 and 18-044).

### 2.3. Doxorubicin Treatment and Cell Transplantation

For the doxorubicin (Dox)-injury model, BL6 male mice aged 12 weeks were injected intraperitoneally with a single dose of doxorubicin hydrochloride (20 mg/kg; Mayne Pharma, Montreal, QC, Canada), or with an equal volume of 0.9% saline, as described earlier [3]. Dox- or saline injected mice (controls) to be used as recipients for systemic cell injection experiments were allowed to recover for three days before cell therapy.

Both NC and RL mice were maintained as homozygous knock-in lines in BL6 background to avoid immune rejection when NCRL cells are injected into wild type BL6 recipient mice. To obtain embryonic ventricular cells for cell injection experiments, the hearts of all NCRL embryos from each pregnant mouse were dissected, atria were moved and the ventricles were dispersed by incubation in 0.2% Type I collagenase for 30–45 min at 37 °C, as reported earlier [23,24]. Following a five-minute interval to allow settling of the fibrous tissue debris, the supernatant was transferred to a new Eppendorf tube and centrifuged (4 min at 4000 rpm, 4 °C). The obtained pellets were resuspended in 10% FBS-DMEM and the centrifugation step was repeated one more time to neutralize the actions of collagenase. The final cell pellets were resuspended in sterile PBS and cell numbers were counted using a hemocytometer. NCRL embryonic ventricular cells were injected into the tail veins of anesthetized recipient mice (0.5 × 10^6^ cells in 30 µL of PBS per recipient). After three- or seven-days post cell injections, recipient hearts were processed for determination of cell transplantation efficiencies as described below.

### 2.4. Assessment of Donor Cell Transplantation Efficiencies

To determine the percentage of cell seeding rates, recipient mice were sacrificed three or seven days after systemic cell injections. The hearts were excised, rinsed in PBS and rocked in Flow Fix (50 mM sodium cacodylate in 1% paraformaldehyde buffer, pH 7.4) for 48 h at 4 °C. A motorized advance Vibroslice system (Model MA752^TM^, Campden Instruments Ltd., Lafayette, IN, USA) was then used to generate 50 µm tissue sections in a PBS tissue bath, and the sections were subsequently rocked overnight at 37 °C in X-Gal solution to elicit a blue chromogenic signal in the transplanted embryonic ventricular NCRL cells. Later, the X-Gal positive tissue sections were placed in 30% sucrose solution in 4 °C overnight, transferred to OCT medium (Sakura Finetek, Torrance, CA, USA) and stored at −80 °C for generating cryosections. Thin sections (10 µm) were generated using a cryostat (Leica CM 3050S, Leica Microsystems Inc., Richmond Hill, ON, USA) and stained with Hoechst 33,258 as described earlier [21]. The donor cell seeding efficiency for each heart was determined by calculating the percentage of X-Gal positive cells out of total number of Hoechst 33,258 positive cells counted from all cryosections.

### 2.5. Immunostaining of Histological Sections

Thin cryostat sections were first hydrated with PBS for 5 min, and fixed with ice cold methanol for 5 min at 4 °C. For sections that were incubated with vWF (Santa Cruz) antibody the Vectastain ABC system (Cat#PK4000, Vector Labs) was used. After fixation, the sections were immersed in 0.3% H_2_O_2_ (hydrogen peroxide in PBS), followed by two consecutive 2-min PBS washes, and then covered in blocking buffer solution (10% *v/v* goat serum (Thermo Fisher Scientific, Waltham, ON, USA), 1% *w/v* BSA (Thermo Fisher Scientific) in PBS) for 5 h at room temperature. Blocking buffer solution was then replaced with blocking buffer containing primary antibodies for Von Willebrand Factor (vWF; Cat# sc-14014; 1:50) overnight at 4 °C. The next day, the slides were washed with PBS three times for 3 min each and immersed in the secondary antibody (75 µL of horse serum, 25 µL of anti-rabbit biotinylated from the Vectstain ABC kit in 5 mL of PBS) for 30 min at room temperature, followed by two consecutive 2-min PBS washes. Next, the sections were immersed in ABC mix (2 drops of solution A and solution B from the kit in 5 mL of PBS) for 30 min at room temperature. Next, the slides were immersed in 3,3′-diaminobenzidine (DAB) solution (1 DAB tablet and 0.375 g of ammonium nickel (II) sulfate in 62.5 mL of 1× TBX (pH adjusted 8.0) and 1 mL of 3% H_2_O_2_ for 2 min. Slides were then subsequently dehydrated in ascending grades of 70%, 90% and 100% ethanol for approximately 2 min and mounted in Cytoseal-60 (Richard-Allan Scientific, Kalamazoo, MI, USA). Blood vessels identified by vWF staining were quantified for each group.

### 2.6. Histochemical Staining Procedures

In order to determine the extent of fibrosis due to Dox treatment, cryosections were processed for Pico Sirius Red and Fast Green staining as previously described [25,26]. In brief, cryosections were initially fixed in 50 mL of Bouin’s solution (35.7 mL of water-saturated picric acid, 1.16 mL of 37% formaldehyde, 10.7 mL of ddH_2_O and 2.41 mL of glacial acetic acid) at 55 °C for 1 h, washed with water, and stained with 0.1% Fast Green (Sigma) for 10 min at room temperature. Next, the sections were rinsed with 0.1% acetic acid for 2 min and stained with 0.1% Sirius Pico Red (Sigma-Direct red 80 in saturated aqueous picric acid) for 30 min at room temperature. Slides were then subsequently dehydrated in ascending grades of 70%, 90% and 100% ethanol, 2 min each, and mounted in Cytoseal-60. Areas occupied by the green and red represent healthy myocardium and fibrotic tissue, respectively.

For hematoxylin and eosin staining procedure, the cryosections were immersed in instant hematoxylin and eosin solutions as per the supplier’s instructions (Thermo Electron Corporation, Pittsburgh, PA, USA) and rinsed with tap water. Sections were subsequently immersed in grades 70%, 90% and 100% ethanol, and xylenes and mounted with Cytoseal-60, and examined by light microscopy using a Leica DM2500 microscope. The hematoxylin stains cell nucleus deep purple-blue and eosin stains the cytoplasm pink. X-Gal stained donor cells were readily visualized after H&E staining in cryosections.

### 2.7. Electrocardiography and Echocardiography

For electrocardiographic studies, animals were anesthetized with 1.5% isoflurane (Pharmaceutical Partners of Canada, Inc., Richmond Hill, ON, Canada). A small animal heating plate (TCAT-2LV^TM^, Physitemp Instruments, Inc., Clifton, NJ, USA) was used to maintain their body temperature at 37 °C, with a rectal probe to monitor the body temperature. The electrocardiogram (ECG) signal was obtained using a bipolar 3-electrode 3-lead system (AD Instruments, Inc., Colorado Springs, CO, USA). The positive and negative leads were placed under the skin of the left and right pectoral muscle and the ground lead was placed under the skin of the left hind limb. ECG signals were recorded using Bio AMP and Power Lab 8/30 hardware. The signal was recorded for 20 min, and at least 10 beats were averaged to determine the heart rate (HR), QRS duration, and the QT, RR, and PR intervals, using Lab Chart 7 v.7.3.7 software (AD Instruments).

For echocardiography studies, animals were anesthetized with 1% isoflurane, and transthoracic echocardiography was performed using a high-resolution transducer and a GE Vivid 7 ultrasound machine (GE Healthcare, Westborough, MA, USA). Cardiac structure and function were assessed by measuring 2-dimensional M-Mode images from the parasternal short axis at the level of midpapillary muscle and the parasternal long axis.

### 2.8. Cell Migration and Invasion Tests

The assessment of cell migration and invasiveness was conducted using the CytoSelect^TM^ Assay kits (Cell Biolabs, San Diego, CA, USA) according to the manufacturer’s instructions for each kit. To obtain pooled ventricular cells, embryonic ventricles from timed- pregnant CD1 mice were collected and then dispersed by incubation in 0.2% Type I collagenase for 30–45 min at 37 °C, as reported earlier [23,24]. The cells were then kept at 37 °C in a 5% CO_2_ incubator during the test.

Cells were analyzed for differences in cell migration using the CytoSelect^TM^ 24-well Cell Migration Assay, as previously described [27]. Briefly, 300 μL of 0.5 × 10^6^ cells/mL serum-free suspensions of pooled embryonic ventricular cells from E11.5 or E14.5 stages were incubated for 22 h at 37 °C in polycarbonate membrane inserts with 8 μm pores, with surrounding wells containing 10% FBS-DMEM. For the analysis of growth factor effects, the wells contained 1% FBS (Low Serum) DMEM and 40 ng/mL of FGF-1 (rMuFGF1^TM^, Gold Biotechnology, St. Louis, MO, USA), its neutralizing antibody (Santa Cruz sc-7910, 100 ng/mL), or both. In the case of PDGF-B, we used rHuPDGFBB^TM^ (20 ng/mL), its antibody sc-7878 (100 ng/mL), or both. Media and non-adherent cells were then removed from the inserts and migratory cells that had passed through the pores were washed and stained with the cell stain solution provided in the kit. The cell stain was subsequently solubilized in the extraction solution, and absorbance was measured at 560 nm.

Cells were analyzed for cell invasiveness using the CytoSelect^TM^ 24-well Cell Invasion Assay, as previously described [27]. Briefly, 250 μL of 0.5 × 10^6^ cells/mL serum free cell suspensions of pooled embryonic ventricular cells at E11.5 or E14.5 were incubated for 24 h at 37 °C in polycarbonate membrane inserts with 8 μm pores and upper surface type I collagen coating, with surrounding wells containing 10% FBS DMEM. Media and non-invasive cells were then removed from the inserts and invasive cells that had passed through the pores were washed and stained as described earlier. The cell stain was solubilized, and absorbance was measured at 560 nm.

### 2.9. RNA Isolation, and Quantitative PCR Amplification

Total RNA was isolated from pooled embryonic ventricular cells at E11.5 or E14.5 (or from adult ventricular tissue) using TRIzol^TM^ reagent (Invitrogen, Burlington, ON, Canada), and reverse-transcribed with the SuperScriptIII^TM^ RT PreMix kit (Invitrogen), according to the manufacturers’ instructions. Real-time quantitative polymerase chain reaction (RT-qPCR) was performed on all samples in duplicate as previously described [24,28]. Amplification of the transcripts used 40 cycles of 15 s at 95 °C and 60 s at 60 °C, with the primers listed in Table 1 (all obtained from Thermo Fischer Scientific, ON) and the EVOlution EvaGreen^TM^ qPCR mix (Montreal Biotech, Quebec City, QC, Canada). DNA levels were subsequently normalized to the RT-qPCR product of a control reference gene, GAPDH (whose expression level we have previously shown to remain unchanged across all developmental and post-natal stages [29], amplified from the same RT reaction, using the ∆∆C*_T_* method [30].

### 2.10. Protein Extraction and Westerns Blotting

To assess the changes in cellular protein content due to Dox treatment, lysates were prepared from the ventricles of the Dox or saline treated animals, and equal amounts of protein were run on 10.5% polyacrylamide gels and transferred to nitrocellulose membranes as previously described [31,32]. Immunodetection of proteins was conducted with the peroxidase-conjugated Fc anti-rabbit IgG (Jackson ImmunoResearch Laboratories, Inc., West Grove, PA, USA; Cat#111-035-046, 1:5000) against rabbit polyclonal FGF-1 (Santa Cruz sc-7910, 1:800), PDGF-B (sc-7878, 1:800), and GAPDH (sc-25778, 1:500). Protein bands were detected by enhanced chemiluminescence using SignalFire^TM^ (Cell Signaling Technology, Danvers, MA, USA) according to the manufacturer’s instructions. Imaging films were then scanned and analyzed by NIH ImageJ software, and the level of each target protein (FGF-1 or PDGF-B) was normalized to that of GAPDH for each sample, as described before [3,24].

### 2.11. Statistical Analysis

Statistical analysis was performed using Graphpad Prism version 7 (Graphpad Software, San Diego, CA, USA). All data were compared using an unpaired Student *t*-test or the ANOVA with Tukey’s multiple comparisons test and are presented as mean ± standard error of the mean (SEM). Differences of *p* < 0.05 were considered significant.

## 3. Results

### 3.1. Embryonic Ventricular Cells Home to Injured Tissue and Increase Angiogenesis

**Figure 1:**
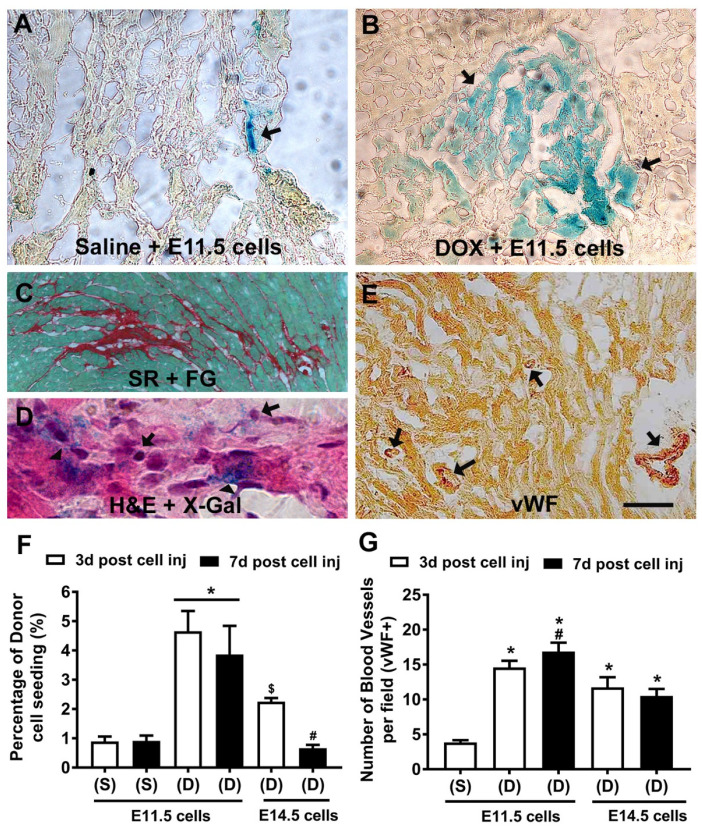
Comparison of homing efficiencies of E11.5 and E14.5 ventricular cells in the host myocardium by tail vein injection method. (**A**–**D**) Representative micrographs from X-Gal stained sections of cell injection experiments. Recipient mice were treated with (**A**) saline or (**B**) doxorubicin (Dox) for 3 days, donor cells were injected 3 days later in the tail veins and recipient hearts were processed after 3 days for X-Gal, or (**C**) Sirius Red and Fast Green (SR + FG) and (**D**) hematoxylin and eosin staining (H&E). Arrows indicate engrafted X-Gal positive donor cells in panels A and B. (**C**) Fibrotic myocardium after Dox treatment is revealed by a red stain. (**D**) Engrafted cells were found frequently in areas of inflammation (arrows indicate small inflammatory cells and arrowheads indicate X-Gal positive donor cells). (**E**) Representative cryosection sequentially processed for von Willebrand factor (vWF) and X-Gal staining. Note absence of X-Gal positive donor cells in blood vessel compartments indicated by the arrows. (**F**) Quantification of engraftment efficiency of X-Gal positive cells in saline (S) or Dox (D) treated mice. * *p* < 0.05 vs. all other groups, $ *p* < 0.05 vs. Dox + 3-days post E11.5 cell injections, # *p* < 0.05 vs. Dox + 3- or 7-days post E11.5 cell injections, One-way ANOVA with Tukey’s multiple comparison test. Results are mean ± SEM of 3 experiments/group. (**G**) Quantification of blood vessels per section in recipient hearts after systemic delivery of donor cells. * *p* < 0.05 saline vs. all other groups, # *p* < 0.05 vs. Dox + 3- or 7-days post E14.5 cell injections and saline group, One-way ANOVA with Tukey’s multiple comparison test. Results are mean ± SEM of 30 sections from 3 recipient hearts/group.

A single intraperitoneal injection of Dox was used to induce myocardial injury as described earlier [3]. Dox-injected mice were allowed to recover for three days and subsequently used as recipients for cell injection experiments. E11.5 and E14.5 ventricular cells obtained from NCRL knock-in mouse embryos [21] were injected in the tail veins of mice treated with saline or Dox. The recipient hearts were harvested 3 or 7 days after cell injections and cryosections were prepared and processed for X-Gal staining (Figure 1A,B). In saline treated recipient hearts, we observed only a limited number of donor cells with E11.5 ventricular cell injections (Figure 1A) but no grafts were found with E14.5 cells (not shown). In contrast, systemic delivery of both E11.5 and E14.5 donor cells led to formation of significantly larger intracardiac grafts in Dox treated mice at 3-day time points (Figure 1B). While the E11.5 graft sizes remained similar at both 3- or 7-day points, the engraftment efficiencies of E14.5 cells were significantly lower than those of E11.5 cells at both time points tested (~2–6-fold difference; Figure 1F). Engrafted cells were frequently found in the areas of Dox-induced injury and inflammation (Figure 1C,D).

We next examined whether engrafted E11.5 or E14.5 ventricular cells could increase new blood vessel formation in recipient hearts. Histological sections were processed for immunostaining with vWF antibodies and X-Gal staining to identify vascular structures in the engrafted areas (Figure 1E). The number of vWF positive vascular structures was significantly higher in Dox treated recipient hearts with E11.5 or E14.5 cell transplants compared to the saline treated control animals (~2–3-fold increase; Figure 1G). Notably, the number of blood vessels per cross sectional area at day 7 was significantly higher with E11.5 cell injections compared to E14.5 cell injections at both time points examined (~1.5-fold increase; Figure 1G).

### 3.2. Quantification of Cell Migration and Invasiveness of Embryonic Ventricular Cells

**Figure 2:**
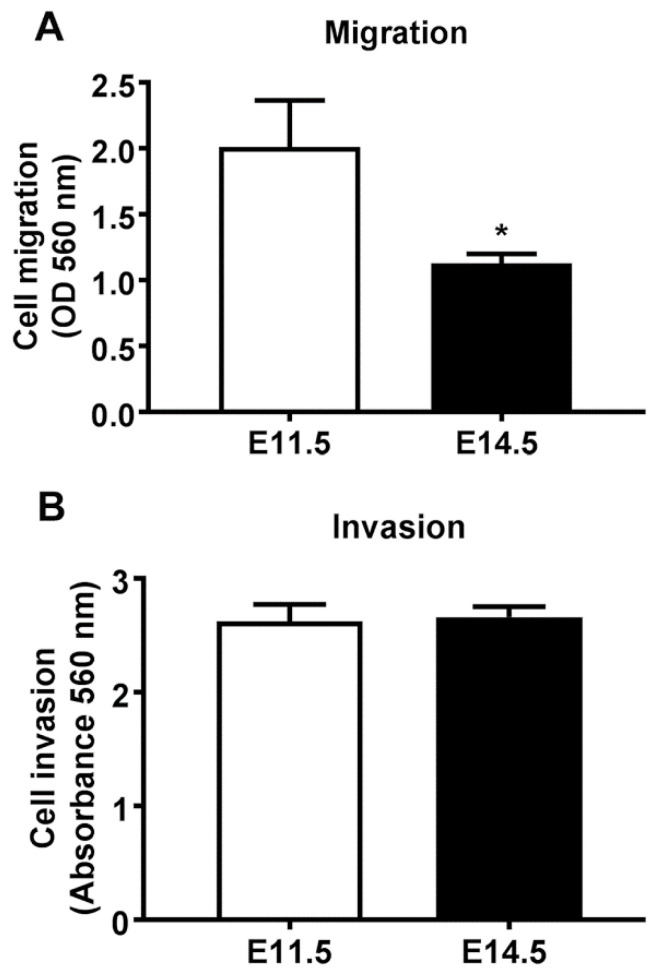
Cell migration (**A**) and invasion (**B**) properties of embryonic ventricular cells. 300 μL of 0.5 × 10^6^ cells/mL serum-free cell suspensions were incubated for 22 h at 37 °C in polycarbonate membrane inserts with 8 μm pores with surrounding wells containing 10% FBS DMEM. The bottom surface of the polycarbonate inserts was coated with type I collagen for the cell invasion assay (**B**), but this coating was absent in cell migration assay (**A**). The inserts were then removed, migratory cells that had passed through the pores were washed and stained, and the cell stain was solubilized for measurement of absorbance. Asterisk indicates that cell migration was significantly lower in the E14.5 cells *p* < 0.05, N = 6 independent experiments for each group).

To explore the characteristics that might foster the greater homing efficiency of E11.5 ventricular cells into the injured heart, we proceeded to examine their behavior in vitro. Cell migration and invasion attributes that may assist in the successful colonization and subsequent repair of cardiac injury sites, were assessed using the CytoSelect^TM^ cell migration and invasion assays. For cell migration assays, serum-free cell suspensions were incubated overnight in membrane inserts (Boyden chambers) with pores that allow cell migration into surrounding wells containing 10% FBS-DMEM. A variation of this assay, in which the inserts were coated with a layer of dried basement membrane matrix solution, permitted only the invasive cells (which can degrade the matrix proteins in that layer) to migrate through the pores. Our findings indicated that the E11.5 ventricular cells have a significantly higher migration capability than that of E14.5 cells (Figure 2A). Although, both E11.5 and E14.5 cells displayed cell invasion properties, there was no significant difference between the two groups (Figure 2B).

### 3.3. Developmental Differences in Growth Factor and Chemokine Receptor Gene Expression in Embryonic Ventricular Cells

**Figure 3:**
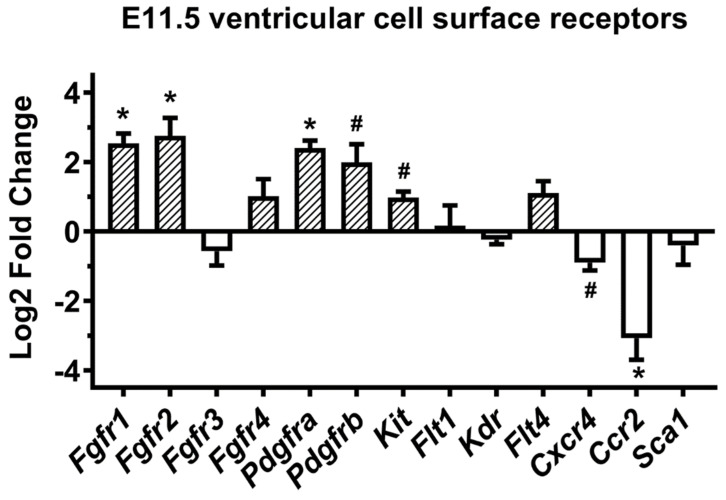
Age-dependent change in cytokine receptor mRNAs in embryonic ventricular cells. Equal amounts of total RNA from E11.5 and E14.5 ventricles were reverse transcribed using oligo-dT primers, and real-time quantitative polymerase chain reaction (RT-qPCR) was used to amplify and quantify the product, with the expression intensity normalized against GAPDH. The log2 fold changes for each transcript in E11.5 vs. E14.5 ventricles were plotted on *y*-axis. * *p* < 0.005 vs. E14.5 ventricles, # *p* < 0.05 vs. E14.5 ventricles. Students unpaired *t*-test. N = 3-4 independent experiments for each group).

E11.5 and E14.5 ventricular cell migration documented under in vitro conditions is likely due to the presence of chemotactic factors present in the surrounding medium (10% FBS-DMEM). Thus, we reasoned that the differential migration rates of E11.5 and E14.5 cells may depend on the differences in their ability to respond to those chemoattractants. We therefore assessed gene expression levels of the surface receptors for chemokines and growth factors known to be vital in cell migration and differentiation during embryonic organogenesis. Equal amount of total RNA from E11.5 and E14.5 ventricles was reverse transcribed, and the cDNA samples were subjected to RT-qPCR with primers specific for selected candidate genes. Gene expression levels were normalized to that of *Gapdh* using ΔΔCT method. The RT-qPCR analysis revealed that several of these candidate receptor mRNAs are expressed at higher levels in the E11.5 ventricles (Figure 3). Specifically, we found that the E11.5 ventricles express significantly higher levels of mRNA for receptors such as *Fgfr1*, *Fgfr2*, *Pdgfra*, *Pdgfrb* and *c-Kit* compared to the levels in E14.5 ventricles (2 to 6-fold differences). Although expression levels of *Fgfr4* and *Flt4* were also higher at E11.5 stage, the difference was not statistically significant (Figure 3). In contrast, we noted lower levels of gene expression for *Fgfr3*, *Kdr*, *Cxcr4, Ccr2* and *Sca1* in E11.5 ventricles, but only *Cxcr4* and *Ccr2* were found to be significantly lower at that stage compared to the levels in E14.5 ventricles (2-fold and 8-fold lower levels respectively, Figure 3).

### 3.4. Assessment of Growth Factor and Chemokine Gene Expression Levels in the Ventricles of Recipient Mice after Treatment with Doxorubicin

**Figure 4:**
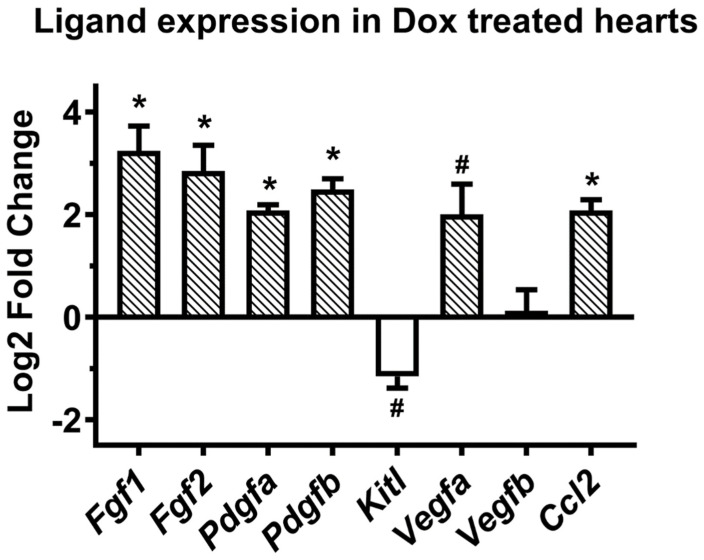
Comparison of chemokine ligand mRNA levels in ventricular cells from adult hearts following doxorubicin treatment. Mice were given intraperitoneal injections of saline (Control) or doxorubicin (Dox), and their hearts were harvested after 3 days. Equal amounts of total RNA were reverse transcribed using oligo-dT primers, and real-time quantitative polymerase chain reaction (RT-qPCR) was used to amplify and quantify the product, with the expression intensity normalized against GAPDH. The log2 fold changes for each transcript in Dox vs. saline ventricles were plotted on *y*-axis. * *p* < 0.005 vs. saline treated group, # *p* < 0.05 vs. saline treated group. Students unpaired t-test. N = 3 independent experiments for each group).

It is possible that the higher homing efficiency of E11.5 ventricular cells in the injured myocardium could be due to increases in certain chemotactic cytokine levels. To test this notion, we used RT-qPCR analysis to assess the gene expression levels of selected chemokines and growth factors in Dox or saline treated hearts. Adult mice were given intraperitoneal injections of saline or Dox, and their hearts were harvested after 3 days. Total RNA was extracted from the ventricles of saline and Dox treated mice and subjected for RT-qPCR analysis using primers specific for selected chemokines and growth factors. Gene expression levels were normalized to that of *Gapdh* using ΔΔCT method. The RT-qPCR analysis revealed that the gene expression levels of ligands such as *Fgf1, Fgf2, Pdgfa, Pdgfb, Vegfa* and *Ccl2* were significantly increased in Dox treated ventricles by day 3 when compared to the expression levels in saline treated ventricles (4- to 9.5-fold differences, Figure 4). No significant changes were found in the gene expression levels of *Vegfb*, whereas *Scf* (Kit ligand) levels were downregulated in Dox treated ventricles compared to those of saline treated ventricles (2.2-fold, Figure 4).

### 3.5. Doxorubicin Treatment Increases FGF1 and PDGFB Protein Levels in the Adult Heart Ventricles

**Figure 5:**
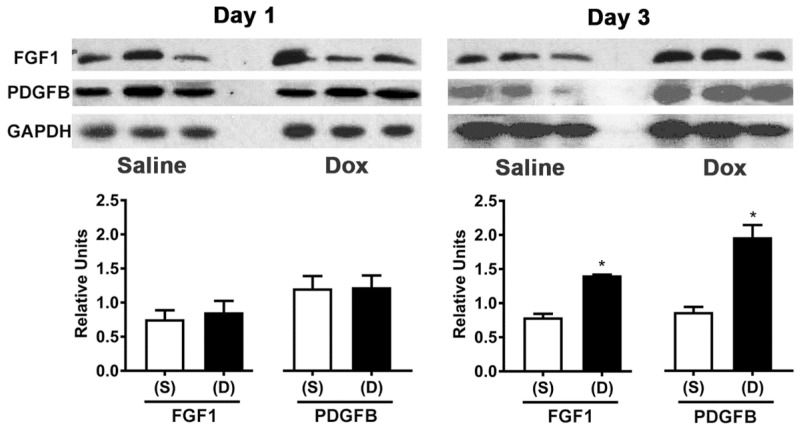
Doxorubicin increases FGF-1 and PDGF-B protein levels in adult heart ventricular cells. Mice were given intraperitoneal injections of saline (S) or doxorubicin (D) and their hearts were harvested after 24 h (Day 1) or 72 h (Day 3). Using Western blotting, lysates containing equal amounts of total protein from the ventricular tissue of the differently treated animals were tested for FGF-1 and PDGF-B protein levels. When standardized against GAPDH, the level of both proteins (FGF-1 and PDGF-B) was found to be significantly higher in the Dox-treated ventricles than in the saline treated ventricles by Day 3. * *p* < 0.05 vs. saline (S), One-way ANOVA with Tukey’s multiple comparisons test, N = 3 independent experiments for each group.

We next evaluated the protein levels of FGF1 and PDGFB in adult hearts at 1- and 3-day time points after treatment with saline or Dox. Tissue lysates containing equal amounts of total protein from the ventricular tissue of saline or Dox treated animals were subjected for Western blotting with antibodies specific for FGF1 and PDGFB. After normalizing to the levels of a loading control (GAPDH), expression levels of both proteins did not change significantly 1-day after Dox treatment when compared to those levels in saline treated ventricles (Figure 5, Day 1). Whereas, the levels of FGF1 and PDGFB were found to be significantly higher in the Dox treated hearts after three days post drug treatment (1.8- and 2.3-fold increases vs. respective saline controls; Figure 5, Day 3).

### 3.6. FGF1 and PDGFB Increase Embryonic Ventricular Cell Migration

**Figure 6:**
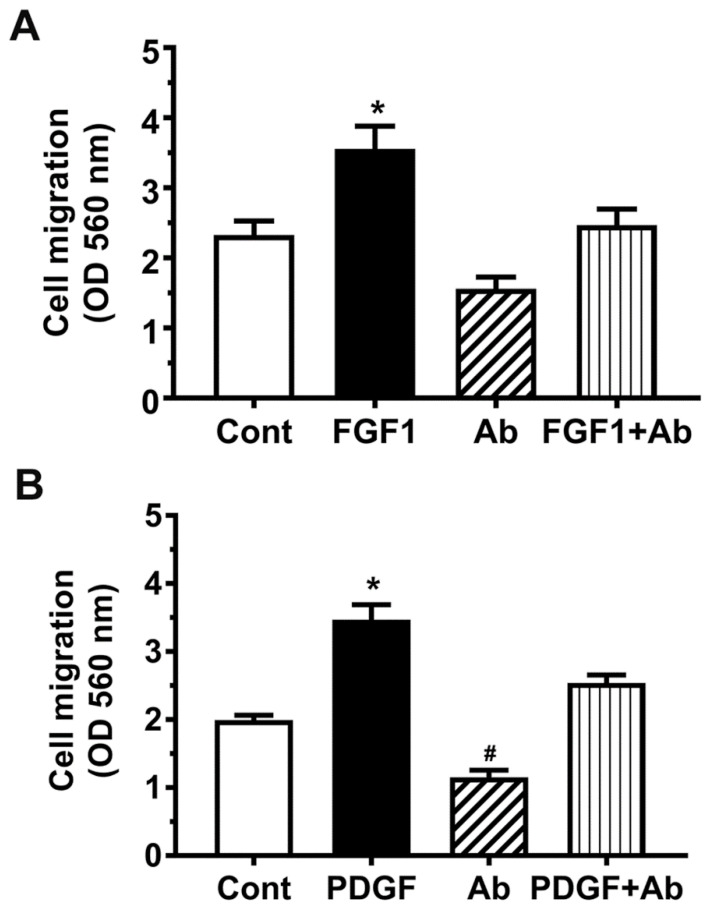
FGF-1 and PDGF-B increase embryonic ventricular cell migration, whereas their neutralizing antibodies decrease migration. 300 μL of 0.5 × 10^6^ cells/mL serum-free cell suspensions were incubated for 22 h at 37 °C in polycarbonate membrane inserts with 8 μm pores, with surrounding wells containing 1% FBS DMEM and FGF-1, its antibody, or both (**A**) or PDGF-B, its antibody, or both (**B**). The inserts were then removed, migratory cells that had passed through the pores were washed and stained, and the cell stain was solubilized for measurement of absorbance. * *p* < 0.05 vs. all other groups; # *p* < 0.05 vs. all other groups, One-way ANOVA with Tukey’s multiple comparisons test, N = 3 independent experiments for each group.

The effects of FGF1 and PDGFB on E11.5 ventricular cell migration, were subsequently examined using the in vitro cell migration assay as described earlier. Serum-free cell suspensions were incubated overnight in membrane inserts (Boyden chambers) placed in surrounding wells containing 1% FBS-DMEM supplemented with or without FGF1 and PDGFB. Our results indicated that exogenous addition of both FGF1 and PDGFB significantly increased the migration of E11.5 ventricular cells compared to the control wells where the medium was not supplemented with these two factors (1.5–1.7-fold, Figure 6A,B). In contrast, the cell migration was inhibited in the presence of neutralizing antibodies specific for FGF1 or PDGFB, but only the effect of PDGFB antibody was found to be significantly different when compared to the control (1.7-fold reduction, Figure 6A,B). In contrast, co-administration of FGF1 or PDGFB was able to prevent the inhibitory effects of neutralizing antibodies on E11.5 cell migration, but those effects were similar to the cell migration observed with respective control groups (Figure 6A,B). These results suggest that both FGF1 and PDGFB can play a chemotactic role in the cell migration of embryonic ventricular cells.

### 3.7. Engraftment of E11.5 Ventricular Cells Can Abrogate ECG Abnormalities and Improve Cardiac Function of the Injured Myocardium

**Figure 7:**
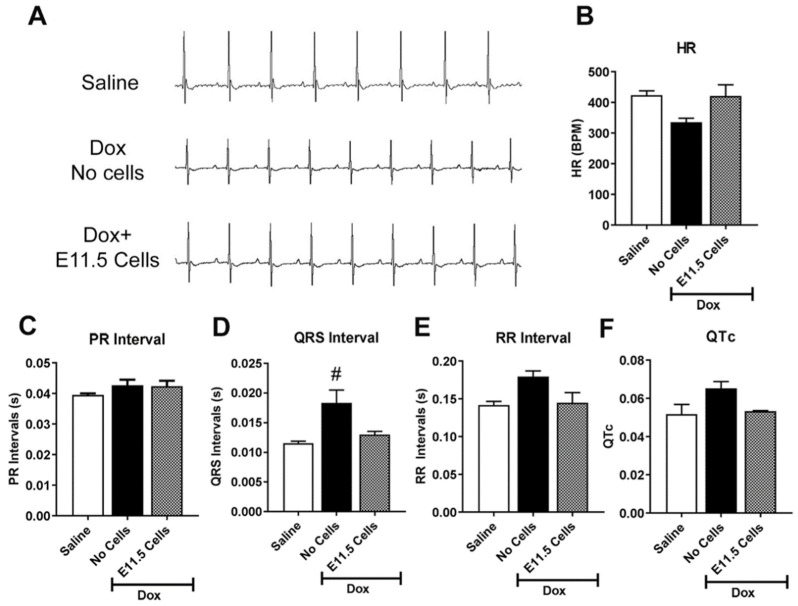
Cardiac function defects can be corrected by embryonic ventricular cell injections. Electrocardiographic analysis of saline or doxorubicin injected C57BL/6 mice treated with or without tail injection of E11.5 ventricular cells. (**A**) Electrocardiogram (ECG) traces of different treatment groups of recipient mice. (**B**–**F**) Quantification of ECG parameters, (**B**) heart rate (HR), (**C**) PR interval, (**D**) QRS interval, (**E**) RR interval, and (**F**) QTc of saline and doxorubicin (Dox)-treated mice injected with and without embryonic day (**E**) 11.5 ventricular cells. (**D**) # *p* < 0.05 vs. other groups, One-way ANOVA with Tukey’s multiple comparison test. Results are mean ± SEM of 3–4 experiments/group. Panels **B**,**C** and **E**,**F**: No significant differences observed between groups.

As we noted a higher engraftment efficiency and a sustained angiogenic response with E11.5 ventricular cells in the injured myocardium, we subsequently used electrocardiography and echocardiography to analyze changes in the cardiac function of Dox-treated mice infused with or without E11.5 ventricular cells. ECG traces were obtained from saline, Dox and Dox + E11.5 cell treated mice 10 days after the initial injection of saline or Dox (Figure 7A; cells were injected three days after the Dox treatment). Quantification of the ECG parameters indicated a significant increase in the QRS interval of Dox-treated mice, compared to the saline treated group and Dox + E11.5 cells treated group (Figure 7D). Although some changes in heart rate, RR interval, PR interval and QTc were evident in the Dox-treated groups with or without cell injections compared to the saline treated group (Figure 7B,C,E,F), these changes were not significant. These results suggest that tail vein infusion of E11.5 ventricular cells was able to normalize the deleterious QRS changes associated with Dox-induced cardiac injury.

**Figure 8:**
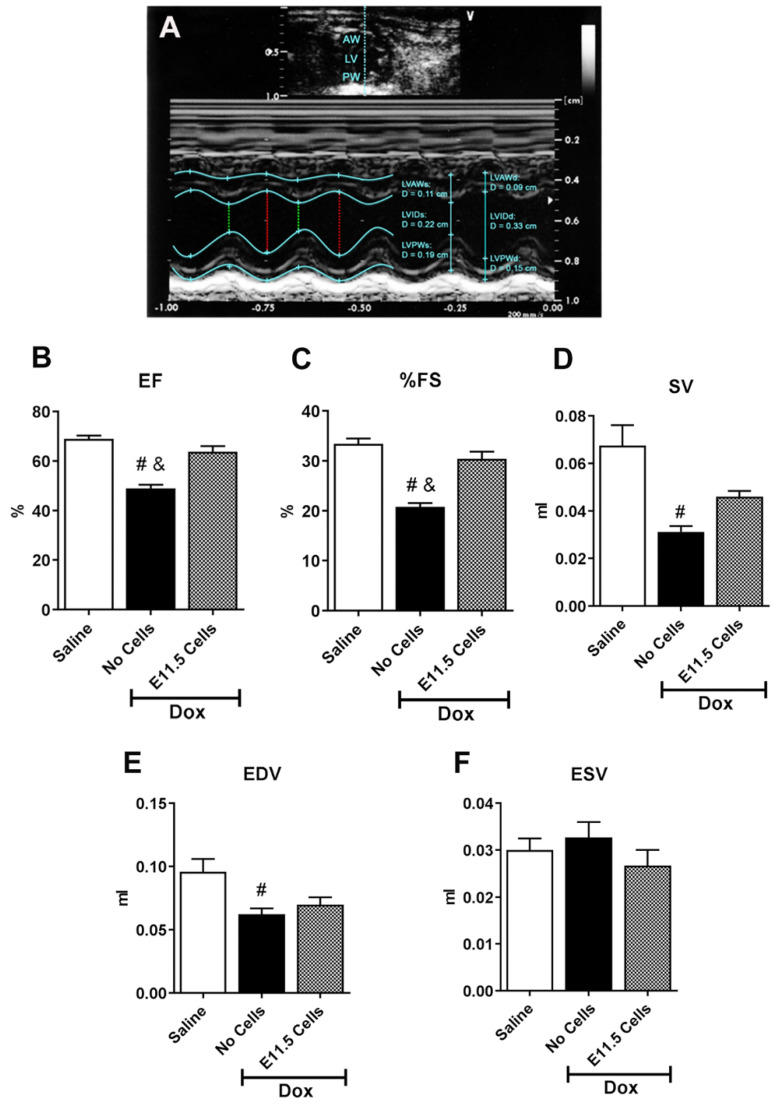
Echocardiographic analysis of Saline or Doxorubicin injected mice treated with or without tail injection of E11.5 ventricular cells. (**A**) Representative short axis M-mode image of left ventricle (LV) illustrating dimensions of LV wall, cavity and cardiac functional measurements. The *y*-axis indicates the distance from transducer (in cm) and the *x*-axis indicates time (in ms). M-Mode images illustrate LV anterior wall (AW) and posterior wall (PW) along with the ventricular chamber, through systole (s; green dotted line) and diastole (d; red dotted line). LVIDs, LV internal diameter during systole; LVIDd, LV internal diameter during diastole. (**B**–**F**) Quantification of short-axis M-mode echocardiogram parameters of untreated and doxorubicin (Dox)-treated BL6 mice treated with and without tail vein injection of E11.5 ventricular cells. (B) EF, ejection fraction, (C) %FS, percent fractional shortening, (**D**) SV, stroke volume, (**E**) EDV, end diastolic volume (**F**) ESV, end systolic volume. # *p* < 0.05 vs. saline, and & *p* < 0.05 vs. Dox + E11.5 cells. One-way ANOVA with Tukey’s multiple comparison test. Results are mean ± SEM of 3–4 experiments/group.

Echocardiography was used to assess the effects of Dox-treatment, by measuring 2-dimensional M-Mode images from the parasternal short axis at the level of midpapillary muscle and the parasternal long axis. Compared to the saline treated group, quantification of the echocardiogram parameters of Dox-treated animals revealed significant decreases in ejection fraction (EF), fractional shortening (FS), stroke volume (SV), end diastolic volume (EDV), LV posterior wall thickness during diastole and systole (LVPWd and LVPWs) and left ventricular internal diameter during diastole (LVIDd) (Figure 8A–E, Table 2), as well as a significant increase in interventricular septum thickness during diastole (IVSd) (Table 2) was evident. Tail vein infusion of E11.5 ventricular cells in Dox-treated animals significantly increased the ejection fraction and percentage of fractional shortening compared to the Dox-treated mice without cell infusions (Figure 8B,C). Although there were some improvements in stroke volume and end diastolic volume parameters of Dox treated mice after E11.5 cell infusion, these values were not significantly different from those of saline or Dox treated mice without cell infusions (Figure 8D,E). Collectively, these results suggest that tail vein infusion of E11.5 ventricular cells was able to normalize structural and functional changes associated with Dox-induced cardiac injury.

## 4. Discussion

We previously showed that direct intracardiac injection of E11.5 ventricular cells can form larger grafts when compared to E14.5 ventricular cells due to developmental differences in the relative distribution of CPCs and CMs and their cell cycle activities [21]. In this study, we further investigated whether there are any differences in the homing efficiencies and functional improvements with the intravenous infusion of embryonic ventricular cells in a Dox induced cardiac injury model.

Dox treatment was shown to cause loss of cardiomyocytes and vascular degeneration [5]. Cardiomyocyte loss after Dox treatment is followed by the appearance of myocardial and perivascular fibrosis as early as 3 days after intraperitoneal injection of a single dose [3]. Migration and homing of mesenchymal stem cells (MSC) to areas of inflammation and tissue injury in animal models of myocardial infarction [33,34,35] and ischemic stroke [36]. However, the role of endogenous stem and progenitor cell recruitment and effects of exogenous cell-based interventions on Dox induced cardiotoxicity have not been fully explored. Although we have not tested the effects of Dox on endothelial cell function and vascular permeability, Dox treatment was shown to increase the endothelial cell permeability by inhibiting the tight junction formation in human cardiac microvascular cells [37]. In a previous study, we have identified the role of p53 independent apoptosis pathways in Dox induced vascular lesions and perivascular fibrosis in the myocardium [3]. Based on these studies, we reasoned that impairment of microvascular barrier function and increased vascular permeability may further facilitate migration and homing of embryonic ventricular cells to the damaged areas in Dox treated hearts.

In this study, a higher engraftment percentage of embryonic ventricular cells was noted in the Dox treated hearts compared to that in saline treated hearts. Notably, the overall cell retention in Dox treated myocardium represented only a small percentage (<3%) of total cells counted in host myocardium and this could be due to cell migration to remote organs such as lungs as was reported with intravenous or intracoronary infusion of MSCs following MI [33,34,38], technical limitations of intravenous cell infusion and cell death within the milieu of injured myocardium [39]. The low cell retention percentage of infused ventricular cells could also be attributed to the lower extravasation into target tissue due to larger cell size (~10–20 μm) or lower expression of cell adhesion molecules which are frequently involved in the transendothelial migration of hematopoietic cells [40]. Differences in cell size and expression profiles of cell adhesion molecules and cell surface receptors involved in cell migration may also account for the different engraftment efficiencies observed with E11.5 vs. E14.5 ventricular cells. Higher migration rate of E11.5 ventricular cells in transwell cell migration assays when compared to that of E14.5 cells is consistent with differences in graft sizes observed with these two cell types in Dox treated hearts.

Factors such as stromal cell derived growth factor-1α, monocyte chemoattractant protein-3 (MCP3) and CC-chemokine ligand-2 (CCL2) were shown to play critical roles in homing of stem cells to the infarcted myocardium [35,41,42]. Notably, Dox induced cardiotoxicity in patients was preceded by increased plasma levels of cytokines/chemokines involved in inflammation [43,44]. Thus, we reasoned that the increased levels of secreted factors in the Dox injury model coupled with the presence of cognate cell surface receptors in donor cells could promote homing of transplanted cells into the heart. To this end, gene expression analysis revealed higher levels of secreted factor receptors (*Fgfr1, Fgfr2, Pdgfra, Pdgfrb* and *c-Kit*) in E11.5 ventricular cells compared to those in E14.5 cells. Whereas, transcript and protein levels for some of these receptor ligands (FGF1 and PDGFB) were upregulated in Dox treated hearts compared to the control levels. In vitro neutralizing antibody experiments further confirmed a regulatory role for FGF1 and PDGFB in donor cell migration in the present study. Our results are in agreement with previous reports which showed that FGF family of growth factors can regulate cell migration during development in multiple organisms [45,46,47]. It has been suggested that FGFs work as chemoattractants such that responding cells become polarized and migrate from areas of low FGF concentration to the source producing higher levels of FGF [45,46]. PDGFB but not PDGFA, was also shown to stimulate chemotaxis in fibroblasts and monocytes [48].

In the present study, the acute Dox induced cardiotoxicity was accompanied by QRS prolongation on ECG and reductions in various cardiac functional parameters monitored by echocardiography. The QRS duration is related to the rate of individual ventricular myocyte depolarization and determined by the time required for intraventricular conduction. While there is scant information on electrocardiographic changes in acute models of Dox induced cardiotoxicity, prolongation of QRS, QT and ST durations were reported for chronic models of Dox treatment in animal studies [49,50] as well as in patients after the first cycle of Dox chemotherapy [4]. While QRS prolongation appears to be one of the early signs of acute cardiotoxicity, changes in QT and ST durations appear to be more reliable indices which correlated well to histopathology in chronic models of Dox induced toxicity [4,49].

Changes in cardiac geometry and cardiac contractility were also monitored using echocardiography. We noted 33% and 26% thinner LVPWd and LVPWs respectively in Dox treated mice 10 days after drug treatment when compared to the saline treated group. These wall thinning effects of Dox could be due to decreases in myofibrillar and cytoskeletal proteins as reported in earlier studies [3,51]. LV wall thinning combined with a 14% reduction in LVIDd also led to significant reductions in EF, FS, SV and EDV in Dox treated mice in this study. Similar changes in cardiac geometry and hemodynamic parameters were also noted in animal models of Dox induced acute cardiotoxicity [52,53,54,55]. For instance, a single injection of Dox (20 mg/Kg) led to significant reductions in LVPWd dimension and fractional shortening (FS) in pregnant rats as early as 48-h time point [52]. In mice subjected to acute Dox treatment (20–25 mg/Kg), significant reductions in LVIDd, LVIDs, FS and cardiac output were noted 4–5 days after drug treatment [54,55].

In this study, a single injection of Dox led to a reduction in LV posterior wall thickness during systole (LVPWs) but an increase in ventricular septum thickness (IVSd) and reduced LV internal diameter (LVIDd) during diastole. These observations contrast with the reports which documented a dilated cardiomyopathy (DCM) phenotype in both patients [56] and animal models [57,58] subjected to chronic Dox treatment alone or in combination with other chemotherapeutic agents. However, there are case reports which did not find a DCM phenotype in patients after Dox treatment. For example, the mean LV diastolic size adjusted for body surface area in pediatric patients subjected for either a single or multiple dose Dox treatment did not differ from that of normal control values [59]. In the same study, LVPW thickness was also lower in Dox treated patients compared to controls [59] and our results are consistent with this report. Whereas, adult FVB/N mice injected with a single dose of Dox did not reveal any significant differences in LVPW, IVS and LVID during systole or diastole while showing significant decreases in the ejection fraction (EF) and % fractional shortening (FS) parameters at 3 and 5 days after acute drug treatment [60]. Another study used multiple low dose Dox injections (7-day intervals) in juvenile DBA/2J mice with the first injection starting at 14 days age and found no significant change in LVIDd over a 17-wk follow up period [61]. Collectively, these studies suggest that DCM phenotype is not always observed in Dox treated animal models or patients and the presence of this phenotype appears to be dependent upon multiple factors such as age, genetic background and the type of Dox treatment (acute vs. chronic) as well as the cumulative dose of Dox.

Despite the absence of a clear DCM phenotype in the present study, reduction in the LVIDd and increased septal wall thickness may indicate the onset of diastolic dysfunction 10 days after Dox treatment. This notion is supported by the reports which showed that a single Dox injection in C57BL/6 mice can lead to significant increases E/E’ ratios at 5 to 12 week time points after drug treatment compared to controls [62,63,64]. The diastolic dysfunction was shown to occur as early as 2 days after Dox injection in a rat model [65]. However, a recent meta-analysis showed that anthracycline-chemotherapy in breast cancer patients without previous cardiac disease had a modest early impact on E/A and E/E’ ratios [66]. Increased levels of left ventricular fibrosis as early as 3 days after Dox treatment in our previous studies [3] warrant further studies on the impact of Dox on diastolic function by measuring mitral valve inflow patterns and tissue doppler velocities at the lateral annulus of the mitral valve. While E11.5 ventricular cell injections normalized reductions in EF and %FS values observed after Dox treatment, cell injections did not have any impact on Dox-induced changes in LVIDd, EDV, IVSd and LVPW thickness. It is likely that increasing the donor cell number or repetitive cell injections at defined intervals may be necessary to normalize these Dox induced hemodynamic and structural changes in the myocardium.

It is possible that cell therapy may have improved cardiac function in Dox treated hearts by preventing the loss of cardiomyocytes and by promoting new blood vessel formation as shown to be the case in several cell transplantation studies [8,9,11]. A significant increase in the number of blood vessels in the recipient hearts after E11.5/E14.5 cell therapy is certainly consistent with the notion that proangiogenic factors secreted from the transplanted cells may be responsible for new blood vessel formation and improvement of cardiac function. Embryonic CPCs were shown to differentiate into cardiomyocytes, endothelial cells and vascular smooth muscle cells [67]. Accordingly, transplanted cells in this study may have undergone spontaneous vascular differentiation or influenced by the local cardiac milieu in Dox treated recipients. Increased levels of FGF1 and PDGFB in Dox treated hearts may have promoted differentiation of transplanted cells to form new blood vessels. Both FGF and PDGF were shown to promote angiogenesis and coronary arteriogenesis [68,69,70]. While the absence of beta-Gal positive cells in the recipient heart blood vessels may rule out the possibility of embryonic CPC differentiation into a vascular cell fate in this study, we cannot rule out the possibility that reporter gene expression may be downregulated after differentiation into a vascular cell lineage. It was shown that E11.5 ventricular cells can secrete both proANP and a biologically active form of 3kD ANP in vitro [24]. Exogenous administration of ANP was shown to inhibit collagen synthesis in cardiac fibroblast cultures and hypertrophy in cardiomyocyte cultures [71,72]. Based on these reports, a similar ANP mediated cardioprotective mechanism may be responsible for functional improvements in Dox treated hearts after E11.5 cell transplantation.

While our studies suggest that E11.5 cells with higher expression of FGF1 and PDGFB receptors are well suited for amelioration of cardiac functional abnormalities in an acute model of Dox cardiotoxicity, further studies should be conducted to determine the efficacy and safety parameters of single and multiple cell injections in chronic models of Dox treatment. Recent reports revealed that exosomes derived from human cardiac resident mesenchymal progenitor cells can attenuate Dox and transtuzumab induced cardiotoxicity [73]. Similarly, extracellular vesicles or exosomes derived from stem or progenitor cell preparations [74,75,76] were shown to improve cardiac function after myocardial infarction. Considering these findings, it would be of great interest to further characterize the secretome of fractionated CPC and CM populations from E11.5 ventricles [77] and further test the effectiveness of E11.5 exosomes in Dox induced cardiotoxicity.

In summary, we found that E11.5 ventricular cells are more efficient in mitigating Dox induced cardiomyopathic effects when compared to E14.5 ventricular cells. Additional in vitro experiments indicated that FGF1 and PDGFB signaling pathways can play a critical role in cell migration and homing of E11.5 ventricular cells in recipient mice after systemic cell injections.

## Figures and Tables

**Table 1:** Primers used for qPCR amplification.

Gene Target	Primer Sequence ^a^, 5′ to 3′	Amplicon Size (bp)
*Fgfr1*	f: AGACTCCACTTCCACAGGGA r: CCAACCTCTAACCGCAGAAC	150
*Fgfr2*	f: GACAAACTCCACATCCCCTC r: ACCACACCTACCACCTCGAT	110
*Fgfr3*	f: CTCCTGCTGGCTAGGTTCAG r: CTCTGGAGCCATGGTAGTCC	145
*Fgfr4*	f: GCTTCATCACCTCCATCTCG r: GTGCAGAGGCCTTTGGTATG	129
*Pdgfra*	f: AGAAAATCCGATACCCGGAG r: AGAGGAGGAGCTTGAGGGAG	95
*Pdgfrb*	f: TGGCCTCTGAGGACTAAAGC r: AACAGAAGACAGCGAGGTGG	118
*Kit*	f: CTCTGATTGTGCTGGATGGAT r: GATCTGCTCTGCGTCCTGTT	111
*Flt1*	f: AAGAGAGTCTGGCCTGCTTG r: CTGCTCGGGTGTCTGCTT	110
*Kdr*	f: GGAAACAGGTGAGGTAGGCA r: AGAGTTGGTGGAGCATTTGG	139
*Flt4*	f: GCTGTCCCCTGCAGGATATG r: GTGGCTCTGCCTCGGACT	131
*Cxcr4*	f: TCCAGACCCCACTTCTTCAG r: AGTGACCCTCTGAGGCGTTT	124
*Ccr2*	f: AGCACATGTGGTGAATCCAA r: TGCCATCATAAAGGAGCCA	91
*Ly6a*	f: GGCAGATGGGTAAGCAAAGA r: CAATTACCTGCCCCTACCCT	108
*Fgf1*	f: GTTGTGATCTCCCCTTCAGC r: CGGACTTCATTCCCGTCTT	123
*Fgf2*	f: TGGCACACACTCCCTTGAT r: AGCGGCTCTACTGCAAGAAC	150
*PdgfA*	f: CCTCACCTGGACCTCTTTCA r: TAACACCAGCAGCGTCAAGT	112
*PdgfB*	f: CAGCCCCATCTTCATCTACG r: CTCTCTGCTGCTACCTGCGT	147
*Kitl*	f: CCGCAGATCTCCTTGGTTT r: GAACAGCTAAACGGAGTCGC	150
*VegfA*	f: AATGCTTTCTCCGCTCTGAA r: CTCACCAAAGCCAGCACATA	126
*VegfB*	f: TGTGCTCCACTCTTCTCCCT r: GGCTTAGAGCTCAACCCAGA	124
*Ccl12*	f: CCTGAAGATCACAGCTTCCC r: GTCCTCAGGTATTGGCTGGA	149
*GAPDH*	f: TCGTCCCGTAGACAAAATGG r: TTGAGGTCAATGAAGGGGTC	132

^a^ f = forward, r = reverse.

**Table 2:** Correction of cardiac structural anomalies by embryonic ventricular cells. Analysis of short-axis M-mode echocardiogram parameters of saline (control) and doxorubicin (Dox) treated C57BL/6 mice injected with or without E11.5 ventricular cells.

	Saline (Control)	Dox-Injected	Dox- Injected + E11.5 Cells
IVSd (mm)	0.83 ± 0.059	0.99 ± 0.034 *	0.87 ± 0.033
LVPWd (mm)	1.47 ± 0.111	1.09 ± 0.034 *	0.87 ± 0.088 *
LVIDd (mm)	3.29 ± 0.132	2.85 ± 0.861 *	2.97 ± 0.881
IVSs (mm)	1.17 ± 0.033	1.21 ± 0.018	1.07 ± 0.089
LVPWs (mm)	1.90 ± 0.102	1.28 ± 0.030 *	1.07 ± 0.0186 *
LVIDs (mm)	2.20 ± 0.070	2.27 ± 0.078	2.17 ± 0.088

IVSd, intraventricular septum during diastole; LVPWd, left ventricular posterior wall during diastole; LVIDd, left ventricular internal diameter during diastole; IVSs, intraventricular septum during systole; LVPWs, left ventricular posterior wall during systole; LVIDs, left ventricular internal diameter during systole. * = significantly different (*p* < 0.05) from saline-injected control. One-way ANOVA with Tukey’s multiple comparison test. Results are the mean ± SEM of 3–5 experiments per group.

## Data Availability

Data and methods used in this paper are presented in sufficient details. Any additional questions should be directed to the corresponding author.
